# Epidemiological and Genetic Data Supporting the Transmission of *Ancylostoma ceylanicum* among Human and Domestic Animals

**DOI:** 10.1371/journal.pntd.0001522

**Published:** 2012-02-07

**Authors:** Romano Ngui, Yvonne A. L. Lim, Rebecca Traub, Rohela Mahmud, Mohd Sani Mistam

**Affiliations:** 1 Department of Parasitology, Faculty of Medicine, University of Malaya, Kuala Lumpur, Malaysia; 2 School of Veterinary Science, The University of Queensland, Gatton, Queensland, Australia; 3 Department of Orang Asli Development, Ministry of Rural and Regional Development, Kuala Lumpur, Malaysia; Fundacao Oswaldo Cruz, Brazil

## Abstract

**Background:**

Currently, information on species-specific hookworm infection is unavailable in Malaysia and is restricted worldwide due to limited application of molecular diagnostic tools. Given the importance of accurate identification of hookworms, this study was conducted as part of an ongoing molecular epidemiological investigation aimed at providing the first documented data on species-specific hookworm infection, associated risk factors and the role of domestic animals as reservoirs for hookworm infections in endemic communities of Malaysia.

**Methods/Findings:**

A total of 634 human and 105 domestic canine and feline fecal samples were randomly collected. The overall prevalence of hookworm in humans and animals determined via microscopy was 9.1% (95% CI = 7.0–11.7%) and 61.9% (95% CI = 51.2–71.2%), respectively. Multivariate analysis indicated that participants without the provision of proper latrine systems (OR = 3.5; 95% CI = 1.53–8.00; p = 0.003), walking barefooted (OR = 5.6; 95% CI = 2.91–10.73; p<0.001) and in close contact with pets or livestock (OR = 2.9; 95% CI = 1.19–7.15; p = 0.009) were more likely to be infected with hookworms. Molecular analysis revealed that while most hookworm-positive individuals were infected with *Necator americanus*, *Ancylostoma ceylanicum* constituted 12.8% of single infections and 10.6% mixed infections with *N. americanus*. As for cats and dogs, 52.0% were positive for *A. ceylanicum*, 46.0% for *Ancylostoma caninum* and 2.0% for *Ancylostoma braziliense* and all were single infections.

**Conclusion:**

This present study provided evidence based on the combination of epidemiological, conventional diagnostic and molecular tools that *A. ceylanicum* infection is common and that its transmission dynamic in endemic areas in Malaysia is heightened by the close contact of human and domestic animal (i.e., dogs and cats) populations.

## Author Summary

Parasitic zoonoses pose a continuing public health problem, especially in endemic developing countries where the majority of populations live in poor, overcrowded conditions, lack education, and practice poor standards of hygiene and improper sanitary disposal of feces. Close contact with domestic animals such as dogs and cats coupled with a lack of proper veterinary care and sanitation expose these communities to high risk of acquiring zoonotic infections. Our study combined the application of conventional and molecular epidemiological tools to determine the probable role of domestic animals, in particular dogs and cats, in the transmission of hookworms in parasite-endemic communities in Malaysia. Epidemiological observation such as univariate and multivariate analyses were used to identify factors associated with hookworm infection in humans, while molecular tools provided the ability to discriminate the different hookworm species, which cannot be achieved by conventional microscopic examination of feces alone. The application of these tools in this present study allowed the accurate determination of parasite species prevalence, hence confirming that *Ancylostoma ceylanicum* infection is common among human population in Malaysia and the close contact of human and domestic animals (i.e., dogs and cats) contributed to the propagation of this hookworm species.

## Introduction

Hookworms are one of the most common parasitic nematodes that inhabit the small intestine of humans and animals such as dogs and cats. The two primary species of hookworm infecting humans are *Ancylostoma duodenale* and *Necator americanus* [1], with *A. duodenale* occurring mainly in the Middle East, North Africa, India, Australia and Europe, whilst *N. americanus* in the Americas, Sub-Saharan Africa, East Asia and Southeast Asia [2]. The socioeconomic and public health impact of human hookworm infections are extensive, infecting an estimated 600 million people worldwide and resulting in up to 135,000 deaths annually [3]. Infection in human causes iron-deficiency anemia which may result in mental retardation and growth deficiencies, particularly in children [4], [5].

Canine and feline hookworm species are also able to cause zoonotic disease in humans. For example, cutaneous larva migrans (CLM) or ‘creeping eruptions’ is a hypersensitivity reaction caused by migrating nematode larvae, of which *Ancylostoma braziliense* is the most frequently implicated aetiological agent in humans [6], . Another canine hookworm, *Ancylostoma caninum* is the leading cause of human eosinophilic enteritis (EE) and an outbreak of 150 cases was reported between 1988 and 1992 in Australia [8]–[10]. Cases have also been reported in the United States [11], Egypt [12], the Philippines, South America and Israel [13].

*Ancylostoma ceylanicum* however, is the only species of animal hookworm known to produce patent infections in humans. This has been demonstrated both experimentally [14], [15] and naturally. Natural infections with *A. ceylanicum* have been reported in Dutch servicemen returning from West New Guinea, who suffered heavy infection with concurrent anemia [16], whilst light infections have been mostly reported from humans in the Philippines [17], Taiwan [18], Thailand [19] and India [20]. More recently, zoonotic ancylostomiasis caused by *A. ceylanicum* was reported in temple and rural communities in Thailand [21], [22] and rural communities in Laos PDR [23] using copro-molecular diagnostic tools. Although hookworm infection is common, especially in rural and remote areas in Malaysia [24], [25], information on the species of hookworms present in humans and domestic animals is currently lacking. Recently, the first data on species-specific hookworm infections was reported by our group [26]. This present study aims to explore the role of domestic animals, particularly dogs and cats as reservoirs for hookworm infection among communities living in rural and remote areas of Malaysia.

## Materials and Methods

### Study area

The study was carried out for a period of two years (i.e., 2009 to 2011) in 8 villages located in remote areas of West Malaysia which were previously recognized as geohelminth-endemic areas [24]. These villages were selected because (i) the hookworm prevalence in these areas were known to be high and (ii) it is accessible by road for rapid transfer of samples to the laboratory. Details of the studied villages and the populations sampled have been described elsewhere [26]. Cats, dogs and poultry are the most common domestic animals. Some villagers had monkeys, rabbits and birds as pets. The majority of these domestic animals are left to roam freely. The villagers have very close contact with the dogs and cats, even sharing food from the same plate with these animals. Occasionally, these animals also slept, defecated indoors and accompanied the villages into the jungle to harvest jungle products.

### Consent, structured questionnaire and fecal sample collection

The aim and procedures of the study were explained and an informed consent sheet was signed by the head of the household or a designated literate substitute. A structured questionnaire was administered to obtain information on the demography (i.e., age, gender, education attainment), socioeconomic (i.e., occupation, household income), behavioral (i.e., personal hygiene such as wearing shoes, defecation practices), as well as information on the environmental sanitation and living condition characteristics such as type of water supply, latrine system and domestic animal ownership or contact. This questionnaire was designed in English and translated to *Bahasa Malaysia*, which is the national language for Malaysia and is well understood by the participants.

Next, a dry, clean and leak proof screw capped pre-labeled fecal container with the individual's name and code was handed out to all participants for the collection of fecal sample the next day. Their ability to recognize their names was also checked. The participants were also guided on how to collect the sample. For the domestic animals, the owners were informed to collect only fresh fecal samples from the defecation site of these animals. Participants who turned up with fecal samples the following day were honored with a small token of appreciation.

### Microscopic examination of fecal samples

The fresh fecal samples were transported back to the Department of Parasitology, Faculty of Medicine, University of Malaya on the same day of collection, preserved in 2.5% potassium dichromate and kept at 4°C until later analysis. Samples were concentrated using formalin ethyl acetate as previously described by Cheesbrough [27] for the presence of hookworms and other intestinal parasites. Briefly, 1 to 2 g of fecal sample was mixed with 7 ml of formalin and 3 ml ethyl acetate, centrifuged, stained with 0.85% iodine and examined under light microscope. Samples which were microscopically positive for hookworm eggs from both humans and animals were further characterized using molecular procedures.

### Genomic DNA extraction

Genomic DNA was extracted directly from positive fecal sample using PowerSoil DNA Isolation Kit (Mo Bio, cat. no. 12888-100, CA, USA) according to the manufacturer's instructions. Briefly, approximately 0.2 to 0.3 g of fecal pellet was added into the PowerBead Tube, incubated at 70°C for 10 minutes with the presence of cell lysis and disruption agent provided by the manufacturer. This were then subjected to homogenization and lysis procedure for complete cell lysis by mechanical shaking (vortexing) using MO BIO Vortex Adapter (MO BIO, cat. no. 13000-V1). The final DNA elution was made in 50 µl of elution buffer and stored at −20°C until required for PCR amplification.

### DNA amplification by PCR

#### Hookworm DNA amplification

A direct PCR assay was used for the DNA amplification of hookworm species in both human and animal samples. Forward primer NC1 (5′-ACG TCT GGT TCA GGG TTC TT-3′) and reverse primer NC2 (5′-TTA GTT TCT TTT CCT CCG CT-3′) [28] were used to amplify an approximately 310 and 420 bp region of the internal transcribed spacer-2 (ITS-2), 5.8S and 28S ribosomal RNA gene of *N. americanus* and *Ancylostoma* spp., respectively. Control samples without DNA (DNase free water, Sigma Cat. no. W4502) and with *N. americanus* and *Ancylostoma* spp. genomic DNA (positive control) were included in each PCR run. The PCR mix consisted of 10× PCR buffer, 2.5 mM dNTPs, 25 mM MgCl_2_, 10 pmol of each primer, 5 U of *Taq* polymerase and 6 µl of DNA template made to a final volume of 50 µl. The sample was heated to 94°C for 5 min, followed by 30 cycles of 94°C for 30 s (denaturing), 55°C for 30 s (annealing), 72°C for 30 s (extension) and a final extension at 72°C for 7 min.

For human samples, an additional PCR assay (two-step semi-nested) was carried out to confirm mixed infection with *N. americanus*. Samples which produced fragments of approximately 310 and/or 420 bp in the first PCR were subjected to a second round of PCR to produce a 250 and/or 130 bp amplicon corresponding to *N. americanus* and *Ancylostoma* spp., respectively. In the second PCR reaction, 6 µl of each NC1-NC2 amplicon was transferred to a fresh tube containing the same PCR reaction buffer with the primer set NA (5′-ATGTGCACGTTATTCACT-3′) for *N. americanus* [29], AD1 (5′- CGA CTT TAG AAC GTT TCG GC-3′) for *Ancylostoma* spp. [30] with NC2 as a common reverse primer and amplified for another 35 cycles. Samples were heated to 94°C for 5 min, followed by 35 cycles of 94°C for 1 min (denaturing), 55°C for 1 min (annealing), 72°C for 1 min (extension) and a final extension at 72°C for 7 min. Cycling was performed in a MyCycler thermal cycler (Bio-Rad, Hercules, USA) for both amplifications.

In order to avoid false negative results via microscopy, all samples found to be negative were also included in the molecular analysis. However, none of these samples were detected positive through PCR.

### Sequencing of PCR product

The positive amplicons were then purified using the QIAquick Gel Extraction Kit (QIAgen, cat. no. 28104, Hilden, Germany) according to the manufacturer's instructions except that final elution of DNA was made in 30 µl of elution buffer instead of 50 µl. All purified amplicons were sequenced in both directions using the same primer sets as in the respective PCR assay with an ABI 3730XL sequencer (Bioneer Corporation, South Korea). Sequence chromatograms were viewed using Sequence Scanner version 1.0 program (Applied Biosystems, USA). Forward and reverse sequences were edited, manually aligned and the consensus sequence was created for each sample using the BioEdit Sequence Alignment Editor program [31]. Similarity searches were carried out using Basic Local Alignment Search Tool (BLAST) to the National Centre for Biotechnology Information (NCBI) reference sequences. All sequences generated in this study were deposited in GenBank, under the accession numbers HQ452515 to HQ452543, JF960362 to JF960403, JN120871 to JN120898 and JN164657 to JN165660.

### Statistical analysis

The data entry and statistical analysis was carried out using the SPSS software (Statistical Package for the Social Sciences) program for Windows version 17 (SPSS, Chicago, IL, USA). Prevalence of hookworm infection in both human and animal samples was determined on the basis of microscopic examination. To describe data, mean and standard deviation for continuous variables and proportion categorical variables were computed. Crude associations of the binary outcome variable with each independent variable were assessed by Pearson's Chi-square (*X^2^*). A univariate model was used to assess potential associations between hookworm infection (outcome of interest) and the potential associated factor characteristics. The level of statistical significance was set at p<0.05 and for each statistically significant factor, an odds ratio (OR) and 95% confidence interval (CI) were computed for both univariate and multivariate logistic regression analysis. All factors that were significant in univariate model were included in a logistic multivariate analysis using backward elimination model to determine which factors could be dropped from the multivariable model.

### Ethical considerations

The study protocol (MEC Ref. No. 824.11) was approved by the Ethics Committee of the University Malaya Medical Centre (UMMC), Malaysia before the commencement of the study. Before participating in the study, parents and their children were given an oral briefing by the investigator on the objective and methodology of the study. Their participation was voluntary and that they could withdraw from the study at any time. They were also informed that their identities and personal particulars will be kept strictly confidential and the procedure used will not pose any potential risk. If they agree to participate, their consent were taken either in written form (signed) or verbally followed by thumb prints (for those who were illiterate) of participants or their parents/guardians (on behalf of their children). For children and very old participants (in the case of incompetent adults), the questionnaire was completed by interviewing their parents and guardians or the relevant adult (normally head of the family) who signed the informed consent.

## Results

### General characteristics and overall prevalence of hookworm infection

Single fecal samples were randomly collected from a total of 634 humans and 105 domestic canine and feline during the study period. Of the 634 human samples, 392 (61.9%) were aged ≤12 years old while 242 (38.1%) aged ≥13 years old with mean age of 18.53±15.89 (mean ± SD). There were 276 (43.5%) males and 358 (56.5%) females. A total of 105 fecal samples were collected from 76 dogs and 29 cats. The overall prevalence of hookworm infection in humans determined via microscopy was 9.1% (58/634; 95% CI = 7.0−11.7%). There were no significant difference in the distribution of hookworm infection among age groups (p = 0.969) and gender (p = 0.729). With regards to domestic carnivores, the overall prevalence of hookworm infections examined microscopically was 61.9% (65/105; 95% CI = 51.2−71.2%). The highest prevalence of hookworm infection was found in dogs (71.1% of 76, 95% CI = 59.5−80.9), followed by cats (37.9 of 29; 95% CI = 20.7−57.7) (Table 1).

**Table 1:** Overall prevalence of hookworm infection in the studied populations as determined by microscopically.

Characteristics	N	n	%	95% CI
**Human samples (N = 634)**				
Gender				
Male	276	24	8.7	5.7–12.7
Female	358	34	9.5	6.7–13.0
Age groups				
≤12 years	392	36	9.2	6.5–12.5
≥13 years	242	22	9.1	5.8–13.4
**Total (Overall prevalence)**	**634**	**58**	**9.1**	**7.0–11.7**
**Animal samples (N = 105)**				
Dogs	76	54	71.1	59.5–80.9
Cats	29	11	37.9	20.7–57.7
**Total (Overall prevalence)**	**105**	**65**	**61.9**	**51.9–71.2**

N: Total number examined; n: Hookworm positive by microscopy; %: Percentage; 95% CI: 95% Confidence interval.

Prevalence of other intestinal parasites detected in these humans were as reported in our previous study [26]. As for domestic carnivores, *Toxocara canis* infection (33/105, 31.4%) was the highest, followed by *Trichuris vulpis* (21/105, 19.7%), *Entamoeba* spp. (13/105, 12.4%), *Giardia* spp. (13/105, 12.4%), *Spirometra* spp. (10/105, 9.5%), *Toxascaris leonina* (5/105, 4.8%), *Balantidium coli* (4/105, 3.8%), *Dipylidium caninum* (4/105, 3.8%) and similar prevalence rates for *Eimeria* spp., *Ascaris* spp. and *Isospora* spp. (2/105, 1.9%), respectively. *Hymenolepis* spp. (1/105, 1.0%) showed the lowest prevalence (data not shown).

### Associated factors for human hookworm infection

Univariate analysis revealed that the lack of a proper latrine system (OR = 2.06; 95% CI = 2.01−2.12; p = 0.015), lack of a pour flush toilet (OR = 2.12; 95% CI = 2.06−2.16; p = <0.001), indiscriminate defecation in the river or bush (OR = 2.10; 95% CI = 2.04−2.13; p = 0.002), walking barefooted outside the house (OR = 2.18; 95% CI = 2.11−2.26; p<0.001), close contact with cats and dogs (OR = 4.52; 95% CI = 1.39−14.72; p = 0.006), eating with hands without prior washing (OR = 2.80; 95% CI = 1.90−7.92; p = 0.043) and using untreated water supply for daily chores (OR = 1.10; 95% CI = 1.02−1.13; p = 0.008) were associated factors for human hookworm infections (Table 2). The final multivariate analysis indicated that participants with inadequate proper latrine were 3.5 times (95% CI = 1.53−8.00; p = 0.003) more likely to be infected with hookworm as compared to those with a proper latrine system in their homes. Walking barefooted had a 5.6 times higher (95% CI = 2.91−10.73; p<0.001) risk of hookworm infection compared to those who wore shoes whilst outdoors. In addition, participants who had close contact with their cats and dogs were 2.9 times more likely to be infected with hookworm (95% CI = 1.19−7.15; p = 0.009).

**Table 2:** Potential associated factors associated with hookworm infection by microscopy in human (Univariate analysis, N = 634).

Characteristics	N	Hookworm		OR (95%CI)	*p* value
		no.	%		
Gender					
Male	276	24	8.7	0.91 (0.53–1.57)	0.729
Female	358	34	9.5	1	
Age (Year)					
≤12 years	392	36	9.2	1.01 (0.58–1.76)	0.969
≥13 years	242	22	9.1	1	
Level of education					
No formal education	244	19	7.8	0.76 (0.43–1.35)	0.347
Formal education	390	39	10.0	1	
Occupational status					
Working	139	14	10.1	1.15 (0.61–2.16)	0.669
Not working	495	44	8.9	1	
Household income (RM/month)					
<RM 500	376	38	10.1	1.34 (0.76–2.36)	0.312
>RM 500	258	20	7.8	1	
Presence of proper latrine system*					
No	341	40	11.7	2.06 (2.01–2.12)	0.015
Yes	293	18	6.1	1	
Type of toilet facility					
None	407	51	12.5	2.12 (2.06–2.16)	<0.001
Pour flush toilet	227	7	3.1	1	
Defaecation places status					
Others (Bush, River)	406	48	11.8	2.10 (2.04–2.13)	0.002
Pour flush toilet	228	10	4.4	1	
Wear shoes/sandals outside the house*					
No	245	45	18.4	2.18 (2.11–2.26)	<0.001
Yes	389	13	3.3	1	
Close contact with cats/dogs*					
Yes	517	55	10.6	4.52 (1.39–14.72)	0.006
No	117	3	2.6	1	
Eat with hand without prior washing					
No	531	54	10.2	2.80 (1.90–7.92)	0.043
Yes	103	4	2.9		
Water supply					
Untreated sources (river, mountain, well)	289	36	12.5	1.10 (1.02–1.13)	0.008
Treated sources (Government pipe)	345	22	6.4	1	
Garbage disposal					
Indiscriminately	330	30	9.1	1.01 (0.59–1.74)	0.958
Collected	304	28	9.2	1	

Reference group marked as OR = 1; 95% CI: 95% Confidence interval; Significant association.

(*p*<0.05).

*Variables were confirmed by multivariate analysis as significant predictors of hookworm infection.

### Molecular characterization of hookworms

Out of 58 human samples found to be positive microscopically, 47 (81.0%) were successfully amplified, sequenced and recently published [26]. Although most hookworm-positive individuals were infected with *Necator americanus*, *Ancylostoma ceylanicum* constituted 12.8% of single infections and 10.6% mixed infections with *N. americanus*.

Out of 65 microscopically positive animal samples, 50 (76.9%) samples were successfully amplified by PCR and sequenced (Table 3). Of these 50 sequences, 52.0% (26 of 50) were found to be 100% homologous to previously published sequences of *A. caninum* (GenBank accession number EU159416, AM850106), 46.0% (23 of 50) samples were 99% identical to *A. ceylanicum* (GenBank accession number DQ438080, DQ831520) and 2.0% (1 of 50) were 100% identical to *A. braziliense* (GenBank accession number DQ438062). All *A. caninum* isolates (26 isolates) were derived from dogs, while 17 dogs (73.9% of 23) and 6 (26.1% of 23) cats were shown to harbor a single *A. ceylanicum* infection. A single *A. braziliense* infection was isolated from cat (data not shown).

**Table 3:** Hookworm species detected from human and animal samples as determined by PCR assays.

Characteristics	PCR and Sequencing	
	n	%
**Human (N = 47)**		
*N. americanus*	36	76.6
*A. ceylanicum*	6	12.8
Mixed (both species)	5	10.6
**Animals (N = 50)**		
*A. caninum* *	26	52.0
*A. ceylanicum* **	23	46.0
*A. braziliense* ***	1	2.0

*A. caninum were found in dogs;

**A. ceylanicum were found in both cats and dogs;

***A. braziliense was found in cats.

## Discussion

To the best of our knowledge, this is the first investigation of the molecular epidemiology of hookworm in both humans and animals living in the same endemic area in Malaysia. Based on microscopic examination of feces, the overall prevalence of hookworm was determined to be at 9.1% in humans and 61.9% in animals (71.1% in dogs and 37.9% in cats). In Malaysia, intestinal parasitic infections (IPIs) including hookworm infections are still a major public health problem, especially among the impoverished and underprivileged communities living in rural and remote areas. This was illustrated by a recent study which reported 73.2% of 716 people were infected with at least one type of soil-transmitted helminth (STH), with 12.8% being hookworm infection [24].

In the present study, factors significantly associated with hookworm infection included lack of provision of proper latrine system, walking barefooted and close contact with cats and dogs. Similar risk factors were also highlighted in previous local studies among rural communities where *A. lumbricoides* and *T. trichiura* infections were high [24], [32], [33]. Observations made in the current study noted that most residents did not have proper sanitary facilities at home. For households that did possess latrines, they were mainly utilized by adults and were poorly maintained. The filth and smell discouraged many from using it. Children were allowed to defecate indiscriminately around their houses. In some cases, even adults defecated indiscriminately in the bushes and nearby river at the back of their houses.

The damp soil and lush vegetation associated with Malaysia's tropical climate form ideal conditions for the development, survival and transmission of hookworm larvae. These conducive environmental conditions coupled with outdoor defecation of humans and animals lead to widespread contamination of hookworm larvae in the soil. Percutaneous infection results when humans walk barefooted outdoors, which is a common practice in this community. Hence, like in previous studies [22], [33] it was not surprising that walking barefooted outdoors constituted a significantly higher risk of being infected with hookworm as compared to those who wore shoes or sandals.

The higher rate of soil-transmitted helminth (STH) infection in this community especially *T. trichiura* and *A. lumbricoides* infections could be due to anthelminthic resistance and high re-infection rates. Currently, the recommended regime for the treatment of STH infections is either albendazole or mebendazole. Although both drugs are deemed broad spectrum anthelminthic agents, important therapeutic differences do exist which affect their uses in clinical practice [34]. While global reports of anthelminthic drug resistance are rare, evidences of emerging resistance do exist throughout the world [34]–[36] and has already been observed and reported in Malaysia [37], [38]. Another important problem encountered in the treatment management is the high re-infection rate occurring especially in highly endemic areas. Two studies in Malaysia have found that re-infection can occur as early as 2 months post treatment, by 4 months almost half of the treated population had been re-infected [37] and by 6 months the intensity of infections had returned to pre-treatment levels [38]. The findings that intensity of STH infections attained the pre-treatment levels by 6 months after treatment have also been reported in other regions of the world [35].

The role of companion animals as reservoirs for zoonotic diseases has been known as a significant health problem worldwide [39]. This current study once again recognizes and highlights the significant association of close contact between humans and animals especially dogs and cats, with regards to hookworm infections. Comparable results were also observed in other rural communities in Malaysia [24], [33] and endemic areas in Thailand [21], [22] and India [40]. In addition, the practice of inappropriate disposal of animal feces in public areas in the studied communities is a common habit among the villagers, leading to environmental contamination and thus increasing the risk of infection to other animals and human.

The probable zoonotic risk of *A. ceylanicum* is of great concern to public health as the hookworm has been shown to be highly endemic among domestic animals in these communities with an overall prevalence of 61.9%. It is difficult to compare the current status of hookworm infection among animals in Malaysia since there is limited prior documented data. Even if data was available, the incrimination of a specific species is not possible as molecular tools were not employed in these previous studies. The only prevalence data on hookworm available was among stray dogs in Kuala Lumpur and Sarawak (East Malaysia) [41], [42] where a rate of more than 95% were reported. More recently, a survey of intestinal parasitic infections (IPIs) of dogs from temples in Thailand revealed that 58.9% of dogs were infected with hookworm [21]. Another survey of IPIs among dogs in rural India also found high prevalence of hookworm ranging from 93.0% to 98.0% [40], [43].

In our molecular analysis, *N. americanus* and *A. ceylanicum* were identified in human, while *A. caninum*, *A. ceylanicum* and *A. braziliense* were detected in animals. Nearly a quarter of hookworm-positive individuals were found to harbor infections with *A. ceylanicum* and 46.0% of hookworm-positive dogs and cats were found infected with this species. This finding implied that dogs and cats may act as the probable sources of infection to humans. The results was further strengthened by our epidemiological analysis which revealed that those individuals who had close contact with cats and dogs were 3 times more likely to be infected with hookworm.

However, as the hookworm species were genetically characterized on the basis of its DNA sequences at the ITS-2, 5.8S and 28S of the ribosomal RNA gene, genetic differences among each species could not be excluded in the present study. The utilization of several and more polymorphic genetic marker than ITS-2 such as cytochrome c oxidase subunit 1 (cox1) gene should be taken into consideration in future study in order to determine if the same genotype of *A. ceylanicum* circulates between the human and animal hosts. In addition, examination of the extent of interspecific sequence differences subsequently would provide phylogentically informative characters to infer the evolutionary relationship of member of the species.

The clinical impact of *A. ceylanicum* in humans remains to be investigated. However, in the present study, all the eleven infected individuals with *A. ceylanicum* were asymptomatic. Unfortunately, the clinical observation of creeping eruptions, and EE, and iron deficiency anemia were not investigated in this study. A recent study on zoonotic ancylostomiasis caused by *A. ceylanicum* in Thailand reported that infected individuals reported to suffer from poor health and abdominal pain [21].The epidemiological and clinical significance of *A. ceylanicum* remains largely unresolved due to the limited availability of published research data. Clinical signs reported ranged from asymptomatic light to heavy infections with anemia, lethargy and excessive hunger [16]. In an experimental infection with *A. ceylanicum* [14], [15], subjects developed clinical signs similar to those described from experimental infection with *N. americanus* and *A. duodenale* in human [44], [45]. Further studies investigating the epidemiology, transmission dynamics and clinical significance of *A. ceylanicum* in a community endemic for hookworm disease will be beneficial in unravelling the true significance of this zoonosis in humans.

Besides *A. ceylanicum*, 46.0% of animals were harboring *A. caninum* and 2.0% with *A. braziliense*. Reports from other countries have highlighted that *A. caninum* is ubiquitously found in dogs and cats in tropical and sub-tropical climates in Asia and Australia [20], [21], [40]–[43], [46]–[48]. To date, there is no reported data on *A. caninum* among dogs in Malaysia. This study demonstrated that all of the eight *A. caninum* isolated were from dogs. This finding was in accordance with previous studies in other countries where *A. caninum* has been reported to be the predominant hookworm species in dogs in Thailand [21], India [40], Australia [46] and many other parts of the world [47]. Although *A. caninum* is regarded as an uncommon parasite of cats, its infection has been reported among cats in Australia [48]. However, none of the cats in this study was infected with *A. caninum*. In addition, BLAST result for one of the hookworm isolated from cat in the present study was 100% identical to a previously published sequence of *A. braziliense*. Malaysia is one of the few countries besides Australia to report on *A. braziliense* infections in the Asia-Pacific region [48].

In conclusion, this present study provided evidence based on the combination of epidemiological, conventional diagnostic and molecular tools that *A. ceylanicum* infection is common and that its transmission dynamic in endemic areas in Malaysia is heightened by the close contact between human and domestic animal (i.e., dogs and cats) populations.

## Supporting Information

Checklist S1 STROBE checklist.
(DOC)
