# Supplementary material for: Epidemiological and Genetic Data Supporting the Transmission of Ancylostoma ceylanicum among Human and Domestic Animals
Source: PLoS Negl Trop Dis. 2012 Feb 7;6(2):e1522. doi: 10.1371/journal.pntd.0001522 (PMC3274503; doi:10.1371/journal.pntd.0001522)
Supplement: Checklist S1 — STROBE checklist. (DOC) [file pntd.0001522.s001.doc]

**Checklist S1: STROBE checklist**

|  | Item No | Recommendation |
| --- | --- | --- |
| **Title and abstract** | 1  2-4 | (*a*) Indicate the study’s design with a commonly used term in the title or the abstract |
|  |  | (*b*) Provide in the abstract an informative and balanced summary of what was done and what was found |
| Introduction | | |
| Background/rationale | 5,6 | Explain the scientific background and rationale for the investigation being reported |
| Objectives | 5,6 | State specific objectives, including any prespecified hypotheses |
| Methods | | |
| Study design | 6 | Present key elements of study design early in the paper |
| Setting | 6 | Describe the setting, locations, and relevant dates, including periods of recruitment, exposure, follow-up, and data collection |
| Participants | 6 | (*a*) Give the eligibility criteria, and the sources and methods of selection of participants |
| Variables | 7-9 | Clearly define all outcomes, exposures, predictors, potential confounders, and effect modifiers. Give diagnostic criteria, if applicable |
| Data sources/ measurement | 7-9 | For each variable of interest, give sources of data and details of methods of assessment (measurement). Describe comparability of assessment methods if there is more than one group |
| Bias | - | Describe any efforts to address potential sources of bias |
| Study size | - | Explain how the study size was arrived at |
| Quantitative variables | 10 | Explain how quantitative variables were handled in the analyses. If applicable, describe which groupings were chosen and why |
| Statistical methods | 10  -  -  - | (*a*) Describe all statistical methods, including those used to control for confounding |
|  |  | (*b*) Describe any methods used to examine subgroups and interactions |
|  |  | (*c*) Explain how missing data were addressed |
|  |  | (*d*) If applicable, describe analytical methods taking account of sampling strategy |
|  |  | (*e*) Describe any sensitivity analyses |
| Results | | |
| Participants | 11  -  - | (a) Report numbers of individuals at each stage of study—eg numbers potentially eligible, examined for eligibility, confirmed eligible, included in the study, completing follow-up, and analysed |
|  |  | (b) Give reasons for non-participation at each stage |
|  |  | (c) Consider use of a flow diagram |
| Descriptive data | 11-12  - | (a) Give characteristics of study participants (eg demographic, clinical, social) and information on exposures and potential confounders |
|  |  | (b) Indicate number of participants with missing data for each variable of interest |
| Outcome data |  | Report numbers of outcome events or summary measures |
| Main results | 12-13  -  - | (*a*) Give unadjusted estimates and, if applicable, confounder-adjusted estimates and their precision (eg, 95% confidence interval). Make clear which confounders were adjusted for and why they were included |
|  |  | (*b*) Report category boundaries when continuous variables were categorized |
|  |  | (*c*) If relevant, consider translating estimates of relative risk into absolute risk for a meaningful time period |
| Other analyses | - | Report other analyses done—eg analyses of subgroups and interactions, and sensitivity analyses |
| Discussion | | |
| Key results | 13 | Summarise key results with reference to study objectives |
| Limitations | 16 | Discuss limitations of the study, taking into account sources of potential bias or imprecision. Discuss both direction and magnitude of any potential bias |
| Interpretation | 14-17 | Give a cautious overall interpretation of results considering objectives, limitations, multiplicity of analyses, results from similar studies, and other relevant evidence |
| Generalisability | - | Discuss the generalisability (external validity) of the study results |
| Other information | | |
| Funding | - | Give the source of funding and the role of the funders for the present study and, if applicable, for the original study on which the present article is based |
